# Empathic accuracy in individuals with schizotypal personality traits

**DOI:** 10.1002/pchj.743

**Published:** 2024-03-26

**Authors:** Ding‐ding Hu, Xiao‐dong Guo, Hong Zheng, Chao Yan, Simon S. Y. Lui, Yan‐yu Wang, Yi Wang, Raymond C. K. Chan

**Affiliations:** ^1^ Neuropsychology and Applied Cognitive Neuroscience Laboratory, CAS Key Laboratory of Mental Health, Institute of Psychology Chinese Academy of Sciences Beijing China; ^2^ Department of Psychology University of Chinese Academy of Sciences Beijing China; ^3^ Key Laboratory of Brain Functional Genomics (MOES & STCSM), School of Psychology and Cognitive Science East China Normal University Shanghai China; ^4^ Department of Psychiatry, School of Clinical Medicine The University of Hong Kong, Hong Kong Special Administrative Region Hong Kong China; ^5^ School of Psychology Weifang Medical University Weifang China

**Keywords:** empathic accuracy, interpersonal dimension, intra‐individual variability, schizotypy, self‐report empathy

## Abstract

Empirical research using the Empathic Accuracy Task (EAT) has suggested that schizophrenia patients and people with schizotypal personality disorder exhibit lower empathic accuracy than healthy people. However, empathic accuracy in a subclinical sample with high levels of schizotypy has seldom been studied. Our study aimed to investigate empathy in a subclinical sample using the Chinese version of the EAT and a self‐report empathy measure. Forty participants with high levels of schizotypy (HS participants) and 40 with low levels of schizotypy (LS participants), as measured by the Schizotypal Personality Questionnaire (SPQ), were recruited. All participants completed the Chinese version of the EAT and the self‐report Questionnaire of Cognitive and Affective Empathy. Empathic accuracy (EA) scores and the intra‐individual variability of EA scores were calculated. Independent samples *t* tests and Pearson correlation analyses were performed to examine group differences in empathy and the relationship between empathy and schizotypy respectively. HS participants exhibited reduced EA for both positive and negative videos, and larger intra‐individual variability of EA for negative videos than LS participants. However, HS and LS participants did not differ in self‐report cognitive empathy. Moreover, the interpersonal dimension of the SPQ was negatively correlated with EAT performance and self‐report cognitive empathy in LS participants. Individuals with HS show poorer performance‐based EA but relatively intact self‐report cognitive empathy. This study provides empirical evidence for the ontogeny of empathy deficits in subclinical populations at risk of developing schizophrenia, supporting early interventions for social cognitive deficits.

## INTRODUCTION

Empathy refers to the ability to recognize, understand, and feel others' emotional states (Preston & de Waal, 2002). Existing evidence supports at least two components of empathy (Reniers et al., 2011; Shamay‐Tsoory, 2011), namely cognitive empathy (i.e., the ability to recognize, understand, and make inferences about others’ emotional states), and affective empathy (i.e., the ability to share emotions and feelings with others). Findings from meta‐analyses suggest that patients with schizophrenia are impaired in both the affective and cognitive components of empathy (Bonfils et al., 2016, 2017).

Schizotypy refers to a latent psychological and personality organization that confers liability to develop psychosis (Meehl, 1962), and it is a useful construct for understanding the psychopathology of the broad psychosis continuum, spanning from clinical to subclinical samples (Kwapil & Barrantes‐Vidal, 2015). Using extreme‐group design, several studies have reported worse cognitive empathy in individuals with high level of schizotypy (Kocsis‐Bogar et al., 2017; Pflum & Gooding, 2018; Zhang et al., 2014), while others have reported non‐significant differences (Aghvinian & Sergi, 2018; Canli et al., 2015; Gooding et al., 2010; Gooding & Pflum, 2011; Jahshan & Sergi, 2007; Leung et al., 2021; Russell‐Smith et al., 2013). Regarding affective empathy, both worse (Pflum & Gooding, 2018; Zhang et al., 2014) and comparable (Aghvinian & Sergi, 2018; Russell‐Smith et al., 2013) scores have been reported in individuals with high levels of schizotypy versus low schizotypy (see Table 1 for a summary). Regarding correlations between empathy and the dimensions of schizotypy measured by the Schizotypal Personality Questionnaire (SPQ; Raine, 1991; see Table 2 for a summary), both cognitive and affective empathy measured by self‐report scales have been repeatedly found to be negatively correlated with the interpersonal dimension of the SPQ. In addition, negative (Eddy & Hansen, 2020; Henry et al., 2008; Nahal et al., 2021) or non‐significant (Aldebot Sacks et al., 2012; Bedwell et al., 2014; Deptula & Bedwell, 2015) correlations have been reported between task performance of cognitive empathy and the cognitive‐perceptual and/or interpersonal dimensions of the SPQ. Taken together, it remains unclear whether and how individuals with high‐level schizotypy are impaired in the distinct components of empathy.

**TABLE 1:** Summary of extreme‐group design studies.

Study	Dichotomization method	Cognitive empathy		Affective empathy	
		Measurements	Group comparison	Measurements	Group comparison
Aghvinian and Sergi (2018)	SPQ‐B	IRI_PT	N.S.	IRI_EC	N.S.
Jahshan and Sergi (2007)	SPQ‐B	TASIT	N.S.		
Kocsis‐Bogar et al. (2017)	SPQ	MASC	High < Low		
Zhang et al. (2014)	SPQ	IRI_PT	High < Low	IRI_EC	High < Low
		GEM_Cognitive	High < Low	GEM_Affective	N.S.
Canli et al. (2015)	MIS	EQ_Total	N.S.		
Gooding et al. (2010)	CPPS	RMET	N.S.		
Gooding and Pflum (2011)	CPPS	RMET	N.S.		
Pflum and Gooding (2018)	CPPS	Derntl Perspective Taking Task	High < Low	Derntl Affective Responsiveness Task	High < Low
Russell‐Smith et al. (2013)	O‐LIFE: UE	RMET	N.S.		
		EQ_Cognitive	N.S.	EQ_Affective	N.S.
Leung et al. (2021)	CPQ‐16/CAPE‐C15/SPQ‐B	RMET	N.S.		

Abbreviations: AE = affective empathy; CAPE = Chinese version of the Community Assessment of Psychic Experiences; CPPS = Chapman Psychosis‐Proneness Scales; CPQ = Chinese version of the Prodromal Questionnaire; EC = empathic concern; EQ = empathic quotient; GEM = Griffith Empathy Measure; IRI = Interpersonal Reactivity Index; MASC = Movie for the Assessment of Social Cognitive; MIS = Magical Ideation Scale; N.S. = non‐significant; O‐LIFE: UE = Oxford–Liverpool Inventory of Feelings and Experiences: Unusual Experience subscale; PT = perspective taking; RMET = Reading the Mind in the Eyes Test; SPQ = Schizotypal Personality Questionnaire; SPQ‐B = Schizotypal Personality Questionnaire‐Brief; TAIST = The Awareness of Social Inference Test.

**TABLE 2:** Summary of correlation analyses between empathy and the SPQ.

Study	Cognitive empathy				Affective empathy			
	Measurements	Relationships with SPQ			Measurements	Relationships with SPQ		
		Cog‐per	Int	Diso		Cog‐per	Int	Diso
Bedwell et al. (2014)	IRI_PT	(+)	(−)	N.S.	IRI_EC	N.S.	(−)	(−)
Henry et al. (2008)	EQ_Cognitive	(+)	(−)	N.S.	EQ_Affective	N.S.	(−)	(−)
Kállai et al. (2019)	IRI_PT	N.S.	(−)	N.S.	IRI_EC	N.S.	(−)	N.S.
Thakkar and Park (2010)	IRI_PT	N.S.	(−)	(−)	IRI_EC	N.S.	(−)	N.S.
Zhang et al. (2014)	GEM_Cognitive	(−)	(−)	(−)	GEM_Affective	N.S.	N.S.	N.S.
Aldebot Sacks et al. (2012)	RMET	N.S.	N.S.	N.S.				
Bedwell et al. (2014)	RMET	N.S.	N.S.	N.S.				
Deptula and Bedwell (2015)	TASIT	N.S.	N.S.	N.S.				
Eddy and Hansen (2020)	RMET	(−)	(+)	N.S.				
Henry et al. (2008)	RMET	(−)	(−)	N.S.				
Nahal et al. (2021)	RMET	(−)	(−)	N.S.				

Abbreviations: Cog‐Per = cognitive‐perceptual; Diso = disorganized; EC = empathic concern; EQ = empathic quotient; GEM =Griffith Empathy Measure; cross sign (+) = Positive correlation; Int = interpersonal; IRI = Interpersonal Reactivity Index; N.S. = non‐significant; PT = perspective taking; minus sign (−) = Negative correlation; RMET = Reading the Mind in the Eyes Test; SPQ = Schizotypal Personality Questionnaire; TAIST = The Awareness of Social Inference Test.

Empathy is usually invoked during social interactions (Shamay‐Tsoory & Hertz, 2022; Zaki, 2014), and hence it is vital to evaluate empathy in a dynamic and naturalistic manner, one involving active engagement rather than passive observation. The concept of Empathic Accuracy (EA) was proposed to measure how accurately an individual can understand others’ emotional states during social interactions using the dyadic interaction paradigm (Ickes, 1993; Ickes et al., 1990). Zaki et al. (2008) developed a standard stimulus paradigm of the Empathy Accuracy Task (EAT), which retained the social interaction features but utilized a series of video clips as stimuli. Participants were asked to continuously rate a target's emotional valence when watching videos in which the target was relating an autobiographical event. Using dynamic, multimodal, real‐life‐like videos as stimuli, the EAT can capture participants' real‐life empathic processing (for a review, see Rum & Perry, 2020). Empirical findings based on the EAT have suggested that patients with schizophrenia exhibited reduced EA compared with healthy controls (Harvey et al., 2013; Kern et al., 2013; Lee et al., 2011, 2013; van Donkersgoed et al., 2019), and that EAT performances were correlated with psychiatric symptoms and social skills in patients with schizophrenia (Olbert et al., 2013). Moreover, patients with schizotypal personality disorder exhibited a lower EA compared with controls (Ripoll et al., 2013). However, few studies have utilized the EAT to investigate empathic ability in individuals with high and low levels of schizotypy.

Our study aimed to apply the EAT to evaluate empathy in individuals with high and low levels of schizotypy as measured by the SPQ. As previous studies reported associations between stronger intra‐individual variability (IIV) and higher scores of schizotypy (Kane et al., 2016; Schmidt‐Hansen & Honey, 2009; Wallace et al., 2019), we also calculated the IIV of EA scores to investigate the stability of EA. In addition, we examined the relationships between schizotypal traits and empathic ability in two groups separately. We hypothesized that: (1) the high level of SPQ (HS) group would show a lower EA and higher IIV of EA, as well as lower scores on self‐report cognitive and affective empathy compared with the low level of schizotypy (LS) group; (2) cognitive‐perceptual and interpersonal dimensions of the SPQ would be negatively associated with EA in both groups; and (3) the interpersonal dimension of the SPQ would be negatively correlated with self‐report empathy in both groups.

## MATERIALS AND METHODS

### Participants

A total of 354 college students completed the SPQ online; participants who scored ≥41 and those below ≤20 were considered as individuals with high and low schizotypy, respectively, based on a large‐scale investigation. As a previous study (Ripoll et al., 2013) found reduced EA in patients with schizotypal personality disorder with a large effect size, we estimated the minimum sample size using G*power software (Faul et al., 2007) with a large effect size (Cohen's *d* = 0.8), *α* = 0.05 and 90% power for a two‐sided independent samples *t* test, resulting in 34 participants in each group. Thus, 40 participants with HS (14 males; mean age 20.53, *SD* = 1.92) and 40 with LS (13 males; mean age 21.15, *SD* = 1.99) were recruited to the current study. All participants were right‐handed, had normal or corrected‐to‐normal vision, and did not have any history of neurological or psychiatric disorders. This study was approved by the Ethics Committee of the Institute of Psychology, Chinese Academy of Sciences (H18030). All participants were informed of the study procedure and signed an informed consent form. Participants were paid 60 RMB (approximately US$10) on completion of the study.

### Measurements

#### The Chinese version of the EAT

A Chinese version of the EAT was developed (Guo et al., 2023; Hu et al., 2022), and details of this paradigm can be found in Supplementary Materials: Data S1. The short version of the Chinese EAT was used in this study, in which eight videos (four positive and four negative) were randomly presented in five different orders to avoid order effects across participants. Participants were asked to watch a video and continuously rate the emotional valence of a target on a 9‐point Likert scale. EA scores were calculated using Spearman's correlation between continuous ratings by the target of each video and by participants and Fisher *r*‐to‐*z* transformed for further analyses. IIVs of EA scores across all videos, positive videos and negative videos for each participant were calculated following a log‐transformation approach (L. P. Wang et al., 2012). After watching each video, participants were asked to rate the perceived target valence (PTV) and perceived target arousal (PTA) on a 9‐point Likert scale, as well as their own emotional feelings, that is, perceiver's self‐valence (PSV) and perceiver's self‐arousal (PSA). Finally, averaged EA scores for all videos, positive videos, negative videos, and averaged emotional ratings (PTV, PTA, PSV and PSA) for positive videos and negative videos were calculated for subsequent analysis.

#### Questionnaire of Cognitive and Affective Empathy

The 31‐item Questionnaire of Cognitive and Affective Empathy (QCAE) (Reniers et al., 2011) comprises five subscales to measure the cognitive and affective components of empathy. Subscales of Perspective Taking and Online Simulation were developed to measure cognitive empathy, while affective empathy was captured by subscales of Emotion Contagion, Proximal Responsivity, and Peripheral Responsivity. The Chinese version of QCAE showed good reliability and validity in both a healthy population (Liang et al., 2019) and a clinical sample with psychiatric disorders (Liang et al., 2020). In our study, Cronbach's alpha coefficients of the whole scale, cognitive, and affective dimensions were .89, .91, and .86, respectively.

#### The Schizotypal Personality Questionnaire

The SPQ (Raine, 1991) is a valid self‐report scale for measuring features of DSM‐III‐R schizotypal personality disorder with a three‐factor model, namely cognitive‐perceptual, interpersonal, and disorganized factors. The revised Chinese version of SPQ has good reliability and validity (Chen et al., 1997). In this study, Cronbach's alpha coefficients were .93, .93, and .92 for the cognitive‐perceptual, interpersonal, and disorganized dimensions, respectively.

### Data analysis

Independent samples *t* tests were used to examine group differences on demographics, EAT performance (including EA scores, IIV of EA scores, PTV, PTA, PSV, PSA for both positive and negative videos), and self‐report scores on empathy scales. Pearson correlation analyses were conducted to examine the relationships between EAT performance, self‐report scores on empathy scales, and the SPQ in the two groups separately. EAT data transformation and calculation of EA scores were performed using Python 3.9.7 (Python Software Foundation, Wilmington, DE, USA). Independent samples *t* tests and correlation analyses were performed using SPSS v22 (IBM Corporation, Armonk, NY). The significance threshold was set at *p* < .05.

## RESULTS

### Demographic information and self‐report empathy scores

The HS and LS groups were comparable in age, years of education, and sex proportion. We did not find any group difference in the affective empathy or cognitive empathy dimensional scores of the QCAE between the HS and the LS group, but a significant difference on the Emotion Contagion subscale was observed, with the HS group scoring higher than the LS group, as Table 3 shows.

**TABLE 3:** Descriptive statistics for the high and low schizotypy groups.

Variables	HS group (*n* = 40)		LS group (*n* = 40)		*t*/*χ* ^2^	*p*	*df*	Cohen's *d* (95% CI)
	Mean	*SD*	Mean	*SD*				
Age (years)	20.53	1.92	21.23	2.08	−1.56	.122	78	−0.35 [−1.59 0.19]
Education (years)	14.88	1.64	15.45	1.99	−1.41	.162	78	−0.32 [−1.39 0.24]
Sex (male: female)	14:26		13:27		0.06	.813		
SPQ_Cog_Per	22.25	4.15	6.80	3.12	18.82**	<.001*	78	4.26 [13.82 17.09]
SPQ_Int	22.15	4.64	5.95	3.70	17.26**	<.001*	78	3.91 [14.33 18.07]
SPQ_Dis	11.03	2.96	2.28	1.50	16.69**	<.001*	78	3.78 [7.71 9.79]
SPQ_Total	50.13	7.10	14.08	5.05	26.18**	<.001*	78	5.93 [33.31 38.79]
QCAE_Cog_Empathy	59.75	8.09	59.97	8.59	−0.12	.904	78	−0.03 [−3.94 3.49]
QCAE_PT	32.92	5.61	33.30	5.21	−0.31	.758	78	−0.07 [−2.79 2.04]
QCAE_OS	26.83	4.11	26.68	4.07	0.16	.870	78	0.04 [−1.67 1.97]
QCAE_Aff_Empathy	32.68	6.72	31.70	5.14	0.73	.468	78	0.17 [−1.69 3.64]
QCAE_EC	12.35	2.64	10.77	1.90	3.06**	.003*	78	0.69 [0.55 2.60]
QCAE_Pro_R	9.20	3.22	9.33	3.03	−0.18	.859	78	−0.05 [−1.52 1.27]
QCAE_Per_R	11.13	2.97	11.60	2.31	−0.80	.427	78	−0.18 [−1.66 0.71]

Abbreviations: Aff_Empathy = affective empathy; Cog_Empathy = cognitive empathy; Cog_Per = cognitive‐perceptual; Dis = disorganized; EC = emotion contagion; HS = high schizotypy; Int = Interpersonal; LS = low schizotypy; OS = online simulation; Per_R = peripheral responsivity; Pro_R = proximal responsivity; PT = perspective taking; QCAE = Questionnaire of Cognitive and Affective Empathy; SPQ = Schizotypal Personality Questionnaire.

* *p* < .05.

** *p* < .01.

### Group differences on task performance of the EAT

**FIGURE 1:**
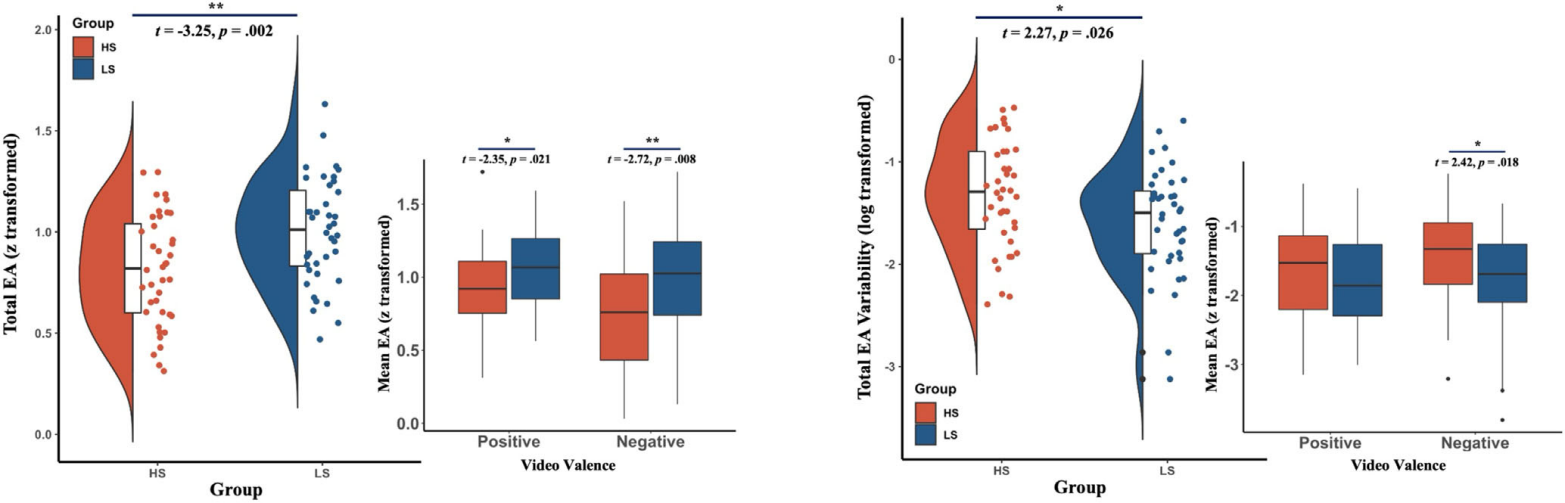
Group comparisons of EA and EA variability between the high and low schizotypy groups. Spearman's correlation coefficient between the continuous emotional valence ratings of the target and the perceiver was calculated as the EA score. Scores of total EA, positive EA, and negative EA are the mean EA scores for all videos, positive videos, and negative videos, respectively. Variabilities of EA scores across all videos, positive, and negative videos for each participant are calculated separately. Independent samples *t* tests were performed to examine group differences on EA and EA variability. The violin plot shows the group differences on total EA or EA variability scores, while the boxplot beside shows the group differences separately for positive and negative videos. EA = empathic accuracy; HS = high schizotypy; LS = low schizotypy.

As Table 4 shows, we found significant group differences in EA scores across all videos, positive videos and negative videos, respectively, with the HS group showing lower EA scores than the LS group. Also, the HS group exhibited a significantly higher IIV of EA scores across all videos and across negative videos than the LS group. As for overall ratings, only marginally significant group differences were found on PTA and PSV of positive videos, with the HS group rated higher on PTA and lower on PSV than the LS group. Among these results, the group difference in EA scores across all videos can survive the Bonferroni correction for multiple comparisons (*p* = .0036 < .05/14) Figure 1.

**TABLE 4:** Group comparisons on the EAT performance.

Task performances		HS group (*n* = 40)		LS group (*n* = 40)		*t*	*p*	*df*	Cohen's *d* (95% CI)
		Mean	*SD*	Mean	*SD*				
All videos	EA^a^	0.81	0.27	1.00	0.26	−3.25**	.002	78	−0.73 [−0.31–0.07]
	Variability of EA^b^	−1.32	0.53	−1.59	0.53	2.27*	.026	78	0.51 [0.03 0.50]
Positive videos	EA^a^	0.92	0.29	1.07	0.28	−2.35*	.021	78	−0.53 [−0.27–0.02]
	Variability of EA^b^	−1.66	0.77	−1.84	0.66	1.11	.273	78	0.25 [−0.14 0.50]
	PTA	6.58	0.99	6.21	0.94	1.71	.091	78	0.39 [−0.06 0.80]
	PTV	7.59	0.74	7.62	0.68	−0.20	.844	78	−0.04 [−0.35 0.28]
	PSA	5.08	1.24	5.03	1.09	0.19	.849	78	0.04 [−0.47 0.57]
	PSV	6.34	0.72	6.65	0.86	−1.76	.082	78	−0.40 [−0.67 0.04]
Negative videos	EA^a^	0.76	0.39	0.99	0.36	−2.72**	.008	78	−0.62 [−0.40–0.06]
	Variability of EA^b^	−1.41	0.66	−1.79	0.76	2.42*	.018	78	0.55 [0.07 0.71]
	PTA	4.74	1.32	4.88	1.11	−0.49	.627	78	−0.11 [−0.70 0.42]
	PTV	2.53	0.89	2.37	0.78	0.84	.406	78	0.19 [−0.22 0.53]
	PSA	4.44	1.43	4.47	1.09	−1.06	.293	78	−0.24 [−0.86 0.26]
	PSV	3.38	0.88	3.10	0.88	1.40	.166	78	0.32 [−0.12 0.67]

Abbreviations: EA = empathic accuracy; EAT = Empathic Accuracy Task; HS = high schizotypy; LS = low schizotypy; PSA = perceiver's self‐arousal; PSV = perceiver's self‐valance; PTA = perceived target's arousal; PTV = perceived target's valance.

* *p* < .05.

** *p* < .01.

^a^ The EA coefficients were Fisher *r*‐to‐*z* transformed.

^b^ The intra‐individual variability of EA was log‐transformed.

### Relationships between empathy and schizotypal traits

**FIGURE 2:**
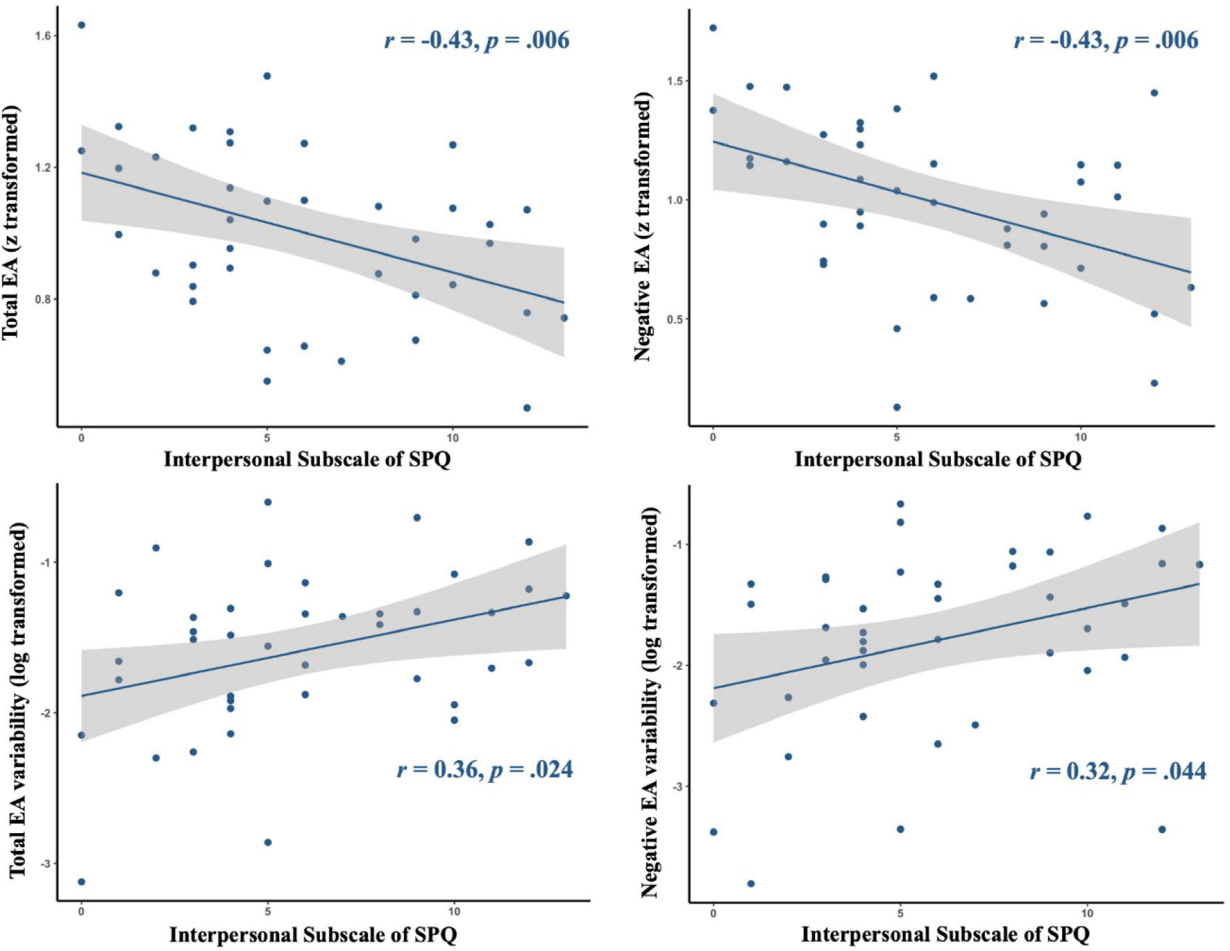
Correlations between EAT performances and the interpersonal subscale of the SPQ in the low schizotypy group. Pearson correlation analyses were performed to examine the relationships between EA or EA variability and the interpersonal subscale of the SPQ in the low schizotypy group. EA = empathic accuracy; SPQ = Schizotypal Personality Questionnaire.

In the LS group, the interpersonal subscale of the SPQ and the total score of the SPQ were negatively correlated with EA and positively correlated with the IIV of EA (Table 5 and Figure 2). In the HS group, a negative correlation was found between the total score of the SPQ and the IIV of EA across positive videos. For the overall ratings, the interpersonal dimension of the SPQ was negatively correlated with PSV after watching positive videos in both groups. The interpersonal dimension of the SPQ was also found to be negatively correlated with PSA and PTA after watching positive videos in the LS group. In addition, the cognitive‐perceptual dimension of the SPQ was positively correlated with PSA after watching negative videos in the LS group.

**TABLE 5:** Correlations between the SPQ and EAT performance empathy scale scores.

Task performance		HS group (*n* = 40)				LS group (*n* = 40)				Whole group (*n* = 80)			
		Cog_Per	Int	Diso	Total	Cog_Per	Int	Diso	Total	Cog_Per	Int	Diso	Total
All videos	EA^a^	0.16	0.24	0.12	0.26	−0.15	−0.43**	−0.21	−0.43**	−0.30**	−0.33**	−0.30**	−0.33**
	Variability of EA^b^	−0.13	−0.17	−0.17	−0.22	0.18	0.36*	0.08	0.35*	0.23*	0.25*	0.18	0.24*
Positive videos	EA^a^	0.18	0.18	0.13	0.27	−0.01	−0.26	−0.15	−0.21	−0.19	−0.23*	−0.21	−0.22*
	Variability of EA^b^	−0.20	−0.27	−0.24	−0.39*	0.02	0.27	−0.04	0.15	0.06	0.09	0.03	0.06
	PTA	0.10	−0.03	−0.00	0.00	−0.05	−0.33*	0.29	−0.12	0.19	0.10	0.21	0.17
	PTV	0.10	0.08	0.12	0.12	−0.08	−0.12	0.18	−0.06	−0.01	−0.02	0.04	−0.01
	PSA	−0.09	−0.25	−0.13	−0.24	−0.05	−0.43**	0.23	−0.19	−0.01	−0.13	0.01	−0.05
	PSV	0.07	−0.33*	−0.20	−0.24	−0.19	−0.33*	0.09	−0.25	−0.20	−0.32**	−0.21	−0.26*
Negative videos	EA^a^	0.08	0.20	0.09	0.17	−0.15	−0.43**	−0.25	−0.44**	−0.27*	−0.29**	−0.27*	−0.30**
	Variability of EA^b^	−0.01	−0.14	−0.21	−0.15	0.15	0.32*	0.25	0.37*	0.27*	0.27*	0.22	0.28*
	PTA	0.08	−0.09	−0.28	−0.16	0.29	0.02	0.03	0.15	0.02	−007	−0.13	−0.07
	PTV	−0.15	−0.16	−0.04	−0.14	0.23	0.11	−0.01	0.21	0.08	0.06	0.07	0.09
	PSA	−0.04	−0.13	−0.20	−0.21	0.44**	0.02	0.08	0.26	−0.05	−0.14	−0.16	−0.13
	PSV	−0.14	0.06	0.03	0.00	0.26	0.16	−0.05	0.23	0.16	0.19	0.14	0.18

Abbreviations: Cog‐Per = cognitive‐perceptual; Diso = disorganized; EA = empathic accuracy; EAT = Empathic Accuracy Task; HS = high schizotypy; Int = interpersonal; LS = low schizotypy; Neg = negative; Pos = positive; PSA = perceived self‐arousal; PSV = perceived self‐valence; PTA = perceived target's arousal; PTV = perceived target's valence; SPQ = Schizotypal Personality Questionnaire; var = variability.

* *p* < .05.

** *p* < .01.

^a^ The EA coefficients were Fisher *r*‐to‐*z* transformed.

^b^ The intra‐individual variability of EA was log‐transformed.

Regarding the associations between self‐report empathy and the SPQ scores, we found that the interpersonal dimension of the SPQ was negatively correlated with the cognitive dimension of the QCAE (*r* = −0.45, *p* = .004), while the disorganized dimension was negatively correlated with the affective dimension of the QCAE (*r* = −0.44, *p* = .005) in the LS group. In the HS group, a significant negative correlation was found between the interpersonal dimension of the SPQ and the affective dimension of the QCAE (*r* = −0.34, *p* = .031).

## DISCUSSION

Our results show that HS individuals exhibited poorer cognitive empathy, indicated by a reduced EA and larger IIV of EA compared with the LS group. Meanwhile, HS individuals did not show significant changes on self‐report cognitive and affective empathy. Moreover, the interpersonal dimension of the SPQ was found to be negatively correlated with EAT performance as well as with self‐report cognitive empathy in the LS group. The interpersonal dimension of the SPQ was also found to be negatively associated with self‐report affective empathy in the HS group.

Previous studies reported impaired cognitive empathy among HS individuals. For example, using the Movie for Assessment of Social Cognition task, HS individuals performed worse than LS individuals when required to answer questions about the characters' feelings after watching a movie (Kocsis‐Bogar et al., 2017; Wastler & Lenzenweger, 2021). In addition, lower EA has been found in clinical samples using the EAT (Harvey et al., 2013; Kern et al., 2013; Lee et al., 2011, 2013; Ripoll et al., 2013; van Donkersgoed et al., 2019). In line with these findings, we found that the HS group exhibited lower EA in the current study, indicating poorer performance‐based cognitive empathy. However, some studies have reported non‐significant group differences between HS and LS groups using experimental tasks, such as the Reading the Mind in the Eyes Test (Gooding et al., 2010; Gooding & Pflum, 2011; Leung et al., 2021). One possible explanation for the inconsistent findings may be related to the paradigms, as HS individuals exhibited deficits only when they had to understand others' feelings during complex situations rather than when encountering simple and static stimuli. In the general population, a previous study suggested that EA was better when auditory rather than visual information was presented (Jospe et al., 2020), suggesting that people may gather information from both sensorimotor cues (such as facial expression) and linguistic cues (such as prosody and semantic information) to better infer others' thoughts and feelings. It is possible that HS individuals might have difficulty in integrating abundant information to understand the feelings of others in complicated everyday life scenarios. Previous studies have suggested abnormal sentence context processing in the auditory modality in individuals with schizotypal personality disorder (Niznikiewicz et al., 1999, 2004). Thus, future research is recommended to separate and combine different kinds of information, such as visual, script, audio, or multimodal versions, in order to unveil the mechanisms of altered empathy in HS individuals.

When relating different dimensions of the SPQ to EAT performance, we found a negative relationship between the interpersonal dimension of the SPQ and EA scores in the LS group, especially for negative videos. However, we did not find similar associations in the HS group. This is understandable, because participants in the LS group may be more representative of the general population. Moreover, previous studies have reported non‐significant relationships between schizotypy and performance‐based empathy in individuals with high levels of schizotypy (Fernyhough et al., 2008; Gooding & Pflum, 2011). Thus, it may be more informative to understand correlations between empathy and schizotypy using a continuum sample rather than extremely high or low groups. For example, previous studies conducted in the general population revealed that cognitive empathy was negatively related to interpersonal dimension of the SPQ (Henry et al., 2008; Thakkar & Park, 2010), or negative schizotypy measured by the Physical Anhedonia Scale and the Revised Social Anhedonia Scale (Guo et al., 2022). Another study using network analysis also indicated direct negative relationships between cognitive empathy and negative schizotypy (Y. Wang et al., 2020). Together with these studies, our findings suggest an important role for the interpersonal dimension of the SPQ on empathic performance.

The trait‐like (which are persistent over time) and state‐like (which fluctuate across different situations) properties for cognitive dysfunction are important (Chan et al., 2022). The IIV is an important and useful variable describing the state‐like behavior among test sessions, trials of a task, or different neuropsychological tasks (Cole et al., 2011). A larger IIV reflects unstable cognitive processing (Ram & Gerstorf, 2009), which cannot be detected by average performance. The previous literature has focused mainly on the IIV among patients with schizophrenia and has reported a larger IIV across trials (Shin et al., 2013) or tasks (Roalf et al., 2013) in this group than in healthy controls. One study (Wallace et al., 2019) tested the IIV of reaction time among university students and found that a larger IIV predicted their psychotic‐like experiences. Partly in line with these studies, we found that the HS group showed a larger IIV of EA than the LS group, especially for negative videos. We found a positive relationship between the IIV of EA and the interpersonal dimension of the SPQ, rather than the cognitive‐perceptual dimension in the LS group. It is plausible that previous studies focused mainly on the IIV of basic cognitive deficits, and that few have examined the variabilities for social and affective processing. Negative schizotypy was measured by the interpersonal dimension of the SPQ in this study, and this construct likely reflects a specific anomaly in social and affective processing (Cohen et al., 2015). More research should be conducted to clarify the relationship between negative schizotypy and the IIV of social cognition.

As for self‐report cognitive empathy, no significant group difference was found, consistent with a previous study using the Interpersonal Reactivity Index and the Empathy Quotient (Aghvinian & Sergi, 2018). Moreover, since we found significant group differences in EA, the inconsistent findings on group differences may suggest that HS individuals exhibit deficits in performance‐based cognitive empathy but lack the corresponding subjective awareness. Similar to our study, Canli et al. (2015) found reduced emotion and face recognition ability in HS individuals, but a non‐significant difference in self‐report empathy. Together with previous studies, our study indicated that social cognition such as empathy may also exhibit subjective–objective disjunction in HS individuals, which might be explained by their lack of awareness of  affective processes (Li et al., 2019; Chan et al., 2011). Although the difference between subjective and objective performances in HS individuals is well recognized (Cohen et al., 2017), previous findings often suggested a more severe subjective dysfunction but an intact objective performance (Chan et al., 2011; Li et al., 2019). Studies can further examine the potential factors contributing to such subjective–objective differentiation, such as cognitive bias or abnormal self‐referential beliefs (Cohen et al., 2017) in HS individuals.

In order to examine empathy from a more comprehensive perspective, we added questions after each video to examine affective empathy but did not find significant group differences. Few studies have examined performance‐based affective empathy in HS individuals. One study using the Affective Responsiveness Task showed less overall accuracy in performance in both the negative schizotypy and the positive schizotypy group than in the control group (Pflum & Gooding, 2018). It is plausible that a deficit in affective empathy may selectively affect different subtypes of schizotypy. For example, Y. Wang et al. (2013) found that participants with negative schizotypy reported significantly lower scores on affective empathy than the low schizotypy group, while participants with positive schizotypy reported similar scores to participants with low schizotypy.

Several limitations of our study should be borne in mind. First, we used the total score of the SPQ to identify HS and LS individuals. Future research could recruit participants using measurements that could differentiate multiple dimensions of schizotypy. Second, although we added questions asking about participants' self‐valence and arousal in the EAT to examine affective empathy, future study could adopt physiological signals or facial expression as more sensitive and objective measures. Third, previous studies indicated that emotional states may have an impact on social cognition processing (Converse et al., 2008; Schmid & Mast, 2010). We did not evaluate the pre‐experiment emotional state of participants, and are thus unable to eliminate the influence of affect.

## CONCLUSIONS

Our findings suggest altered cognitive empathy measured by EA and its IIV in HS individuals compared with LS individuals, and highlight the important role of the interpersonal dimension of the SPQ on empathic performance. These findings imply that individuals with high levels of schizotypy may have difficulty in understanding the emotions of others when encountering complicated everyday social scenarios, which may further lead to negative experiences in social interactions and impede their day‐to‐day interpersonal communication. Our findings provide empirical evidence for the ontogeny of altered social cognition in clinical and subclinical populations, highlighting the need for early interventions.

## CONFLICT OF INTEREST STATEMENT

The authors declare no conflict of interests.

## ETHICS STATEMENT

The current study was approved by the Ethics Committee of the Institute of Psychology, Chinese Academy of Sciences (H18030).

## Supporting information


**Data S1.** Supporting Information.
