# Supplementary material for: Empathic accuracy in individuals with schizotypal personality traits
Source: Psych J. 2024 Mar 26;13(5):813–23. doi: 10.1002/pchj.743 (PMC11444733; doi:10.1002/pchj.743)
Supplement: Supplementary file 1 — Data S1. Supporting Information. [file PCHJ-13-813-s001.docx]

**Supplementary Material**

1. **The Development and Validation of the Chinese Version of the Empathic Accuracy Task (EAT)**

The Chinese version of EAT was developed based on the Standard Stimulus Paradigm of the Empathic Accuracy Task (Zaki et al., 2008).

In the **development phase**, we videotaped 15 ‘target’ participants (mean age = 27 years, SD = 9.62; 8 females) who provided four most positive and four most negative autobiographical events while being videotaped. Then, we played the videos of themselves to the targets and asked them to rate their emotional valence continuously on a 9-point Likert scale (1 = *very negative*; 9 = *very positive*) by pressing arrow keys to move a slider. After playing each video, the targets were asked to rate the overall valence (1 = *very negative*; 9 = *very positive*) and arousal (1 = *Extremely calm*; 9 = *Extremely aroused*) of the video on a 9-point Likert scale. We sought the targets’ authorization for use of these videos, which would then be played in the following experiment. The authorized videos of the targets were then assessed by a panel of four psychologists to ensure that the video materials met the following criteria: (1) having an overall valence ≠ 5 or arousal rating ≥ 5, (2) contents that are clearly expressed by the target, and (3) having different contents across the videos. At last, we chose 9 positive (6 with male targets) and 9 negative videos (2 with male targets), each lasted 1-4 minutes, as the finalized Chinese version of the EAT (see **Supplementary Table S1**).

In the **Validation phase,** 57 university students (25 males; mean age 20.56, SD = $\pm$0.64 years) were recruited as ‘perceivers’ to clarify the reliability and validity of the Chinese version EAT. During the Chinese version of EAT, perceivers were asked to watch videos and continuously evaluate the target’s emotion valence on a 9-point Likert scale (1 = *Very negative*; 9 = *Very positive*) by moving a slider. After each video, perceivers rated the overall emotion valence and arousal of the target in the video on a 9-point Likert scale. Therefore, we collected (1) the perceived target’s valence (PTV, 1 = *Very negative*; 9 = *Very positive*), (2) the perceived target’s arousal (PTA, 1 = *Extremely calm*; 9 = *Extremely aroused*), (3) the perceiver’s self-valence (PSV, 1 = *Very negative*; 9 = *Very positive*), and (4) the perceiver’s self-arousal (PSA, 1 = *Extremely calm*; 9 = *Extremely aroused*). In addition to EAT, we also administered the Questionnaire of Cognitive and Affective Empathy (QCAE; Reniers et al., 2011) and the Interpersonal Reactivity Index (IRI; Davis et al., 1980).

Performances on the EAT were measured by *Empathic accuracy (EA)* and *Overall ratings on emotion valence and arousal*. First, the affective-rating time-series were interpolated and averaged every two seconds. The Spearman correlation coefficient between the targets’ rating time-series and perceivers’ rating time-series of the targets was calculated as the EA score for the perceiver of this specific video. Finally, the mean EA scores for all videos, positive videos, and negative videos were calculated on a participant-by-participant basis. All EA coefficients were Fisher *r*-to-*z* transformed before statistical analyses. In addition, the average ratings of the PTV, PTA, PSV, PSA for positive videos and negative videos were calculated respectively.

The results showed that our EAT has good **internal consistency** (Cronbach’s alpha >0.7 for all behavioral variables (EA, PTV, PTA, PSV, PSA) except for EA score of negative videos (alpha=0.69). Regarding **test-retest reliability**, 36 (17 males; mean age = 20.59±0.57) university students completed the EAT twice in a two-month interval, and we found test-retest reliability coefficients of >0.5 except for EA scores for positive (0.47) and negative videos (0.44). Regarding **criterion validity**, the EA scores, PTV and PTA were significantly correlated with the cognitive dimension of the empathy scales. Moreover, PSV and PSA were significantly correlated with self-report scores on affective empathy. Details are summarized in the **Supplementary Tables S2-S3.**

**References**

Davis, M. H., Davis, M. P., Davis, M., Davis, M., Davis, M., Davis, M., Davis, M., Davis, F. C., Davis, H. A., & Davis, I. W. (1980). A Multidimensional Approach to Individual Differences in Empathy.

Reniers, R. L., Corcoran, R., Drake, R., Shryane, N. M., & Vollm, B. A. (2011). The QCAE: a Questionnaire of Cognitive and Affective Empathy. *Journal of Personality Assessment*, *93*(1), 84-95. https://doi.org/10.1080/00223891.2010.528484

Zaki, J., Bolger, N., & Ochsner, K. (2008). It takes two: the interpersonal nature of empathic accuracy. *Psychological Science*, *19*(4), 399-404. https://doi.org/10.1111/j.1467-9280.2008.02099.x

### Supplementary Tables

**Table S1. Information of Videos**

| Video ID | Target’s gender | Video Valance | Duration (s) | Target’s Valance | Target’s Arousal | Item-total correlation (*r*)^b^ | Item-total correlation (*r*)^c^ |
| --- | --- | --- | --- | --- | --- | --- | --- |
| 07B122^a^ | Male | Negative | 232.439 | 1 | 8 | 0.66 | 0.71 |
| 07B924^a^ | Male | Negative | 131.159 | 2 | 6 | 0.61 | 0.65 |
| 17F571^a^ | Female | Negative | 154.199 | 2 | 7 | 0.58 | 0.61 |
| 12F755^a^ | Female | Negative | 176.759 | 1 | 7 | 0.32 | 0.42 |
| 17F370 | Female | Negative | 223.319 | 1 | 9 | 0.58 | 0.57 |
| 12F553 | Female | Negative | 138.839 | 3 | 7 | 0.43 | 0.39 |
| 17F767 | Female | Negative | 270.839 | 1 | 9 | 0.29 | 0.30 |
| 12F154 | Female | Negative | 130.199 | 2 | 6 | 0.64 | 0.60 |
| 17F372 | Female | Negative | 188.736 | 2 | 8 | 0.57 | 0.67 |
| 08B236 | Male | Positive | 241.559 | 8 | 7 | 0.55 | 0.55 |
| 05B412 | Male | Positive | 198.839 | 7 | 6 | 0.62 | 0.72 |
| 05B611 | Male | Positive | 171.959 | 6 | 6 | 0.31 | 0.39 |
| 05B814 | Male | Positive | 107.639 | 7 | 6 | 0.15 | 0.28 |
| 11B650^a^ | Male | Positive | 90.839 | 7 | 6 | 0.45 | 0.54 |
| 05B813^a^ | Male | Positive | 135.959 | 8 | 7 | 0.45 | 0.49 |
| 17F273^a^ | Female | Positive | 211.272 | 8 | 8 | 0.63 | 0.74 |
| 12F451^a^ | Female | Positive | 96.552 | 7 | 6 | 0.59 | 0.75 |
| 17F274 | Female | Positive | 164.759 | 1 | 9 | 0.51 | 0.53 |

**Notes:**

a. videos that are included in the short version of the Chinese version of Empathic Accuracy Task.

b: the Pearson correlation between the Empathic Accuracy (EA) of each video and the mean score of the EA of all videos.

c: the Pearson correlation between the EA of each video and the mean score of the EA of all videos with the corresponding valence (e.g., if the video is a positive video, then the item-total correlation of the specific video is the Pearson correlation between the EA of this video and the mean score of the EA of all positive videos).

**Table S2. Reliability of EAT**

| Indexes of EAT | | Internal consistency (Cronbach's α) | Test-retest reliability(*r*) |
| --- | --- | --- | --- |
| Negative Videos | EA | 0.69 | 0.44 |
|  | PSA | 0.86 | 0.53 |
|  | PSV | 0.75 | 0.72 |
|  | PTA | 0.87 | 0.75 |
|  | PTV | 0.79 | 0.46 |
| Positive Videos | EA | 0.72 | 0.47 |
|  | PSA | 0.89 | 0.69 |
|  | PSV | 0.89 | 0.66 |
|  | PTA | 0.88 | 0.66 |
|  | PTV | 0.87 | 0.66 |

**Notes:** EAT = Empathic Accuracy Task; EA = Empathic Accuracy; PTA = Perceived Target’s Arousal; PTV = Perceived Target’s Valance; PSA = Perceiver’s Self-Arousal; PSV = Perceiver’s Self-Valance.

**Table S3. Correlations between EAT and self-report empathy**

| Indexes of EAT | | Cognitive Empathy | | Affective Empathy | |
| --- | --- | --- | --- | --- | --- |
|  |  | IRI_PT | QCAE_Cog | IRI_EC | QCAE_Aff |
| Cognitive Empathy | Neg_EA | 0.10 | **0.32^*^** | -0.08 | -0.26 |
|  | Neg_PTA | 0.15 | 0.16 | -0.06 | -0.01 |
|  | Neg_PTV | -0.19 | -0.24 | -0.15 | 0.08 |
|  | Pos_EA | 0.07 | **0.37^**^** | 0.05 | -0.04 |
|  | Pos_PTA | 0.10 | **0.27^*^** | 0.26 | 0.19 |
|  | Pos_PTV | 0.11 | **0.30^*^** | 0.20 | 0.20 |
| Affective Empathy | Neg_PSA | 0.20 | 0.23 | 0.04 | 0.05 |
|  | Neg_PSV | -0.22 | -0.12 | **-0.33^*^** | -0.12 |
|  | Pos_PSA | 0.24 | **0.34^*^** | **0.33^*^** | 0.13 |
|  | Pos_PSV | 0.05 | 0.21 | **0.31^*^** | 0.22 |

**Notes:** EAT = Empathic Accuracy Task; EA = Empathic Accuracy; PTA = Perceived Target’s Arousal; PTV = Perceived Target’s Valance; PSA = Perceiver’s Self-Arousal; PSV = Perceiver’s Self-Valance; Pos = Positive; Neg = Negative; IRI = Interpersonal Reactivity Index; QCAE = Questionnaire of Cognitive and Affective Empathy; PT = Perspective Taking; EC = Empathic Concern; Cog = Cognitive; Aff = Affective.

^**^*p* < 0.01; ^*^*p* < 0.05.
